# Thyroid, cortisol and growth hormone levels in adult Nigerians with metabolic syndrome

**DOI:** 10.11604/pamj.2017.26.52.9909

**Published:** 2017-01-31

**Authors:** Ifeoma Christiana Udenze, Olusola Festus Olowoselu, Ephraim Uchenna Egbuagha, Temitope Adewunmi Oshodi

**Affiliations:** 1 Department of Clinical Pathology, College of Medicine, University of Lagos, Nigeria; 2 Department of Haematology and Blood transfusion, College of Medicine, University of Lagos, Nigeria

**Keywords:** Thyroid hormone, cortisol, growth hormone, metabolic syndrome

## Abstract

**Introduction:**

The similarities in presentation of cortisol excess, growth hormone deficiency, hypothyroidism and metabolic syndrome suggest that subtle abnormalities of these endocrine hormones may play a causal role in the development of metabolic syndrome. The aim of this study is to determine the levels of cortisol, thyroid and growth hormones in adult Nigerians with metabolic syndrome and determine the relationship between levels of these hormones and components of the syndrome.

**Methods:**

This was a case control study conducted at the Lagos University Teaching Hospital, Lagos, Nigeria. Participants were fifty adult men and women with the metabolic syndrome, and fifty, age and sex matched males and females without the metabolic syndrome. Metabolic syndrome was defined based on the NCEP-ATPIII criteria. Written Informed consent was obtained from the participants. Socio demographic and clinical data were collected using a structured questionnaire. Venous blood was collected after an over-night fast. The Ethics committee of the Lagos University Teaching Hospital, Lagos, Nigeria, approved the study protocol. Comparison of continuous variables was done using the Student's t test. Correlation analysis was employed to determine the associations between variables. Statistical significance was set at P<0.05.

**Results:**

Triiodotyronine (T3) was significantly decreased (p<0.001) and thyroxine (T4 ) significantly increased ( p<0.001) in metabolic syndrome compared to healthy controls. T3 correlated positively and significantly with waist circumference (p=0.004), glucose (p= 0.002), total cholesterol ( p=0.001) and LDL- cholesterol ( p<0.001 ) and negatively with body mass index ( p<0.001 )and triglyceride ( p=0.026). T4 had a negative significant correlation with waist circumference (p=0.004). Cortisol and growth hormone levels were similar in metabolic syndrome and controls. Cortisol however had a positive significant correlation with waist/hip ratio (p<0.001) while growth hormone correlated positively with HDL ( p=0.023)and negatively with diastolic blood pressure (p=0.049).

**Conclusion:**

Thyroid hormones T3 and T4 were associated with metabolic syndrome. The thyroid hormones, cortisol and growth hormones correlated with components of the syndrome. A therapeutic role may exit for these hormones in the management of metabolic syndrome and related disorders.

## Introduction

Disorders of some endocrine hormones have features similar to the components of the metabolic syndrome and this has led to the suggestion that a mild degree of endocrine disorders may underlie the metabolic syndrome phenotype [1–3]. These endocrinopathies include disorders of cortisol, thyroid and growth hormone secretion [1–3]. Cushing's syndrome is an endocrine disorder characterised by excess cotisol levels in blood and increased urinary cortisol excretion [4]. Cortisol is a glucocorticoid hormone secreted by the zona glomerulosa cells of the adrenal cortex in response to adrenocorticotrophin hormone secretion from the pituitary gland [5]. The primary function of cortisol is in the physiological adaptation to stress [6]. Through its action on glucose and lipid metabolism, cortisol ensures adequate provision of metabolic substrates to cope with stressful conditions [6]. Adrenocortical hyperfunction arising from tumours of the adrenal cortex lead to Cushing's disease and cortisol excess [6]. Cortisol hyperactivity mediated through an increased adipose tissue mass produces clinical features similar to metabolic syndrome [7, 8]. Cross-sectional studies have shown that individuals with raised blood pressure, glucose intolerance or other features of the metabolic syndrome have raised fasting plasma cortisol concentrations [9, 10]. Growth hormone promotes protein synthesis, lipid degradation and energy metabolism, where, acting as a counter regulatory hormone, interacts with insulin to modulate its control of carbohydrate metabolism [11]. A relationship between growth hormone and cortisol has been described [12]. Growth hormone has an inhibitory effect on the 11beta-hydroxysteroid dehydrogenase enzyme 1, responsible for activating cortisol from inactive cortisone in the liver and adipose tissue, hence growth hormone deficiency effectively increases cortisol production in key target tissues of liver and adipose tissue, promoting insulin resistance and visceral adiposity [12]. Occult abnormalities of the thyroid gland, notably subclinical hypothyroidism [2] and the sick euthyroid syndrome [13] have also been linked with increased prevalence of the metabolic syndrome and its components. The criteria defining metabolic syndrome according to the National Cholesterol Education Programme-Adult Treatment Panel III (NCEP-ATPIII) [14] require a combination of at least 3 of the following 5 criteria: Abdominal circumference ≥ 102 cm in males or ≥ 88 cm in females, HDL cholesterol < 1.03 mmol/L (< 40 mg/dL) [males] or < 1.3 mmol/L (< 50 mg/dL) [females], triglycerides ≥ 1.7 mmol/L (≥150 mg/dL), blood pressure ≥ 130/85 mmHg or the patient receiving hypotensive treatment and fasting glycaemia > 6.1 mmol/L (> 110 mg/dL). The prevalence of metabolic syndrome has increased greatly not only in industrialized nations but also in developing countries, reaching pandemic proportions [15–17]. The treatment modality so far is the control of the individual components of the syndrome to prevent the occurrence of cardiovascular complications or progression to type 2 diabetes [18]. It is unclear whether a single endocrine abnormality triggers the cascade of events that leads to the manifestation of the metabolic syndrome. An understanding of related or associated mechanisms in metabolic syndrome may change management strategies and lead to improved outcomes. Clinical trials using preparations of cortisol synthesis inhibitor such as ketoconazole and recombinant growth hormone are still on going in white populations [19, 20]. There are few studies investigating the role of cortisol, thyroid and growth hormones in adult Nigerians with metabolic syndrome. This study aims to determine the levels of cortisol, thyroid and growth hormones in adult Nigerians with metabolic syndrome and determine the relationship between levels of these hormones and components of the syndrome.

## Methods

This is a cross sectional study of fifty adult men and women with metabolic syndrome and fifty, age and sex matched males and females without metabolic syndrome. The Ethical Research and Review Committee of the hospital approved the study protocol and informed consent was obtained from the participants. The study participants were patients attending The Obesity and Metabolic Clinic of the Lagos University Teaching Hospital. Adult men and women between the age of 30 and 70 years who agreed to participate in the study were consecutively recruited. Socio demographic and clinical data were obtained from the participants using a structured questionnaire. Anthropometric measurements such as weight, height, waist and hip circumference and blood pressure readings were taken. Lipid profile results were also determined. The diagnosis of the metabolic syndrome was based on the NCEP-ATPIII criteria [14]. Subjects who didn't meet the criteria for metabolic syndrome were matched for age and sex with the cases and recruited as controls. The inclusion criteria included adult males and females between 30 and 70 years of age who had any three of the following: Abdominal circumference ≥ 102 cm in males or ≥ 88 cm in females, HDL cholesterol < 1.03 mmol/L (< 40 mg/dL) (females) or < 1.3 mmol/L (< 50 mg/dL) [females], triglycerides ≥ 1.7 mmol/L (≥ 150 mg/dL), blood pressure ≥ 130/85 mmHg or the patient receiving hypotensive treatment and fasting glycaemia > 6.1 mmol/L (> 110 mg/dL) [14]. Persons with diabetes and pregnant women were excluded from the study. The study participants reported on the morning of the study after an overnight (10-12 hours) fast. 5 millilitres of venous blood was collected from the ante cubital vein and transferred into plain tubes for lipid profile, growth hormone, cortisol and free T4, free T3 and TSH assays and into fluoride oxalate tubes for glucose analysis. Abdominal obesity was determined by measurement of the waist circumference. The measurement was taken at the end of several consecutive natural breaths, at a level parallel to the floor, midpoint between the top of the iliac crest and the lower margin of the last palpable rib in the mid axillary line [21]. The hip circumference was measured at a level parallel to the floor, at the largest circumference of the buttocks [21]. The blood pressure was determined using the Accoson's Mercury Sphygmomanometer (cuff size 15×43cm). The subjects were seated and rested for 5 minutes before measurement. The systolic blood pressure was taken at the first Korotkoff sound and diastolic at the fifth Korotkoff sound [22]. The total, LDL, HDL cholesterol and triglyceride were determined on fasting serum samples and glucose concentrations were determined from fasting fluoride oxalate plasma using reagents from Randox Laboratories Limited, Antrim, UK, BT 29 4QY, on semiautomatic biochemistry analyser BS3000P-Sinnowa Medical Science and Technology Company limited, Nanjing, China (211135). Serum levels of growth hormone, cortisol, free T4, free T3, and TSH were determined using reagents from Biovendor Laboratories, 62100 Brno, Czech Republic by an enzyme linked immunoassay technique [23] on Acurex Plate Read - Acurex Diagnostics, Ohio, USA (419-872-4775). The data were analysed using the IBM SPSS version 20.0 package. Independent student's t test was employed to test the differences in the mean values for the continuous variables. Spearman's correlation analysis was employed to determine the association between variables. Statistical significance was set at p < 0.05.

## Results

Fifty subjects met the criteria for metabolic syndrome and were recruited as cases. Another fifty subjects without metabolic syndrome were matched for age and sex with the cases and recruited as controls. The study population included twenty men and thirty women with metabolic syndrome and age and sex matched controls.Table 1 shows the age, sex and ethnic distribution of the study participants. The mean age of the cases with metabolic syndrome was 47.16±13.4 years and 46.79±12.7 years for the controls. Two-thirds of the study participants were between 30-50 years of age, two-thirds were females and majority belonged to the Yoruba tribe. The age and sex matched cases and controls however did not differ in their age, sex and ethnic characteristics. Table 2 shows the demographic characteristics of the study participants. Over sixty percent of the study participants had tertiary education, over seventy percent were Christians and most were married. The study participants did not differ in their demographic characteristics. Table 3 shows the nature of work and lifestyle habits of the study participants Almost eighty percent of the study participants had sedentary lifestyles though majority neither smoked nor took alcohol. The study participants did not differ in their lifestyle habitsTable 4 shows the clinical and laboratory characteristics of the study participants. There was a statistically significant difference in the diastolic blood pressure values between the groups with and without metabolic syndrome. The various measures of obesity also differed significantly between the two groups. Of the lipid profile parameters, only HDL cholesterol differed significantly between the groups. Table 5 shows the levels of the endocrine hormones in subjects with and without metabolic syndrome. Free T3 was significantly lower and free T4 significantly higher in the metabolic syndrome group compared to controls. The group with metabolic syndrome had higher cortisol and lower growth hormone values than the control subjects though the differences were not statistically significant. Table 6 shows the correlation of the endocrine hormones with components of the metabolic syndrome Free T3 had a direct relationship with waist circumference, glucose, total cholesterol and LDL cholesterol and an inverse relationship with BMI and triglyceride. T3 had an inverse relationship with waist circumference. Growth hormone correlated positively with HDL and negatively with diastolic blood pressure while cortisol has a strong direct correlation with waist/hip ratio

**Table 1:** Age, sex and ethnic distributions of the study participants

Characteristics	Metabolic syndrome N=50 (%)	No metabolic syndrome N=50(%)	P value
**Age (mean±sd)**	47.16±13.4	46.79±12.7	0.92
**Age group(years)**			
30-50	30(60)	30(60)	1.0
51-70	40(40)	40(40)	
**Sex**			
Females	30(60)	30(60)	1.0
Males	20(40)	20(40)	
**Ethnicity**			
Yoruba	29(58)	34(68)	0.567
Igbo	15(30)	11(22)	
Others	6(12)	5(10)	

**Table 2:** Demographic characteristics of the study participants

Characteristics	Metabolic syndrome N=50 (%)	No metabolic syndrome N=50 (%)	P value
**Level of education**			
Primary	0(0)	1(2)	0.60
Secondary	17(34)	15(30)	
Polytechnic/University	33(66)	34(68)	
**Religion**			
Christianity	43(86)	39(78)	0.297
Islam	7(14)	11(22)	
**Marital status**			
Married	44(88)	39(78)	0.183
Single	6(12)	11(22)	

**Table 3:** Lifestyle habits and nature of work of the study participants

Characteristics	Metabolic syndrome N=50 (%)	No Metabolic syndrome N=50 (%)	P
**Nature of work**			
Sedentary	40(80)	39(78)	0.806
Non Sedentary	10(20)	11(22)	
**Smoking**			
No	45(90)	49(98)	0.159
Stopped	2(4)	1(2)	
Yes	3(6)	0(0)	
**Alcohol**			
No	34(68)	36(72)	0.082
Occasional	5(10)	10(20)	
Yes	11(22)	4(8)	

The age and sex matched cases and controls did not differ in their lifestyle characteristics

**Table 4:** Clinical and laboratory parameters of the study participants

Parameters	Metabolic syndrome (N=50)	No Metabolic syndrome (N=50)	P
**Age (years)**	48.32±6.62	47.84±6.43	0.807
**SBP(mmHg)**	131.61±16.36	124.53±19.70	0.817
**DBP(mmHg)**	83.43±11.42	76.00±13.56	0.049+
**WC(cm)**	99.11±9.35	89.00±12.54	0.003+
**BMI(kg/m^2^)**	30.59±4.64	24.57±10.90	0.013+
**WHR**	0.88±0.04	0.84±0.54	0.024+
**Glucose(mmoles/L)**	4.88±1.20	4.75±2.79	0.829
**TG(mmoles/L)**	1.92±0.12	1.83±0.22	0.110
**HDL(mmoles/L)**	1.26±0.12	1.36±0.13	0.012+
**TC(mmoles/L)**	5.08±0.46	5.20±0.40	0.367
**LDL(mmoles/L)**	2.94±0.44	2.99±0.43	0.663

+ statistically significant, SBP-systolic blood pressure, DBP-diastolic blood pressure, WC-waist circumference, WHR-waist/hip ratio

**Table 5:** The levels of the endocrine hormones in subjects with and without metabolic syndrome

Hormones	Metabolic syndrome (N=50)	No Metabolic syndrome (N=50)	P
freeT_3_(pmoles/L)	2.88±1.24	5.09±2.37	< 0.001+
freeT_4_(pmoles/L)	17.31±10.06	11.49±2.82	< 0.001+
TSH(miU/L)	1.03±0.88	1.18±0.97	0.446
Cortisol (µg/dl)	12.80±4.79	10.83±6.59	0.437
Growth hormone(ng/ml)	0.42±0.42	0.57±0.62	0.297

+ statistically significant, T_3_-triiodothyronine, T_4_-thyroxine

**Table 6:** Correlation of the endocrine hormones with components of the metabolic syndrome

Hormones	MS components	Spearman’s corr coefficient	P
**Triiodothyronine(T_3_)**	WC	0.306	0.004+
	BMI	-0.217	0.019+
	Glucose	0.281	0.002+
	TG	-0.206	0.026+
	TC	0.299	0.001+
	LDL	0.345	< 0.001+
**Thyroxine(T_4_)**	WC	-0.0306	0.004+
**Growth hormone**	HDL	0.331	0.023+
	DBP	-0.589	0.049+
**Cortisol**	WHR	0.489	< 0.001+

+ statistically significant

## Discussion

This study reports a statistically significant decrease in serum free triiodotyronine (T3) and a statistically significant increase in serum free thyroxine (T4 ) in people with metabolic syndrome compared to healthy controls, suggesting that some degree of thyroid hypofunction is associated with metabolic syndrome [2, 13]. A study from Nigeria by Ogbera et al; [24] reported a prevalence of 40% for metabolic syndrome in patients with hypothyroidism. In our study, T3 also correlated with most of the features of metabolic syndrome, T3 correlated positively with waist circumference, glucose, total cholesterol and LDL- cholesterol and negatively with body mass index and triglyceride. A systematic review by Iwen et al; [25] showed convincing evidence for a major impact of thyroid function on all components of the metabolic syndrome. Thyroid dysfunction is associated with changes in body weight and composition, body temperature, and basal metabolic rate [26]. Both subclinical and overt hypothyroidism are frequently associated with weight gain while hyperthyroidism has been associated with weight loss due to catabolic effects on muscle and adipose tissue [25] explaining the inverse relationship between T3 and BMI reported in this study. In this study, waist circumference, a surrogate marker of abdominal obesity, correlated positively with T3 and negatively with T4. This may be because leptin, an adipokine which functions through the hypothalamus to decrease adipose tissue mass, activates thyroidal 5'deiodinase enzyme, increasing the conversion of T3 from T4 [27, 28]. Thyroid hormones play important roles in the regulation of glucose and lipid metabolism [27]. In the fasting state, thyroid hormones, together with other counter insulin hormones stimulate hepatic gluconeogenesis resulting in increased blood glucose [29].

Thyroid hormones affect many of the enzymes involved in cholesterol synthesis and lipid metabolism which explains findings from this study. Thyroid hormones induce the 3-hydroxy-3-methylglutaryl-coenzyme A (HMG-CoA) reductase, which is the first step in cholesterol biosynthesis. T3 upregulates LDL receptors by controlling the LDL receptor gene activation, this it does by the direct binding of T3 to specific thyroid hormone responsive elements [30]. T3 also controls the sterol regulatory element-binding protein-2, which in turn regulates LDL receptor's gene expression [31]. Furthermore, T3 up-regulates apolipoprotein AV, which plays a major role in TG regulation with the effect of decreasing TG levels by decreasing hepatic production of triglyceride from VLDL [32, 33]. Thyroid hormones can also influence HDL metabolism by increasing cholesteryl ester transfer protein activity [34]. In this study, cortisol and growth hormone levels were not significantly different in the groups with and without metabolic syndrome, although they correlated with some features of metabolic syndrome. A similar study in obese children and adolescents [35] reported a weak association between cortisol and metabolic syndrome components. A study in South Asians [36] reported comparable cortisol results between metabolic syndrome and healthy controls but reported strong associations between cortisol and most of the components of metabolic syndrome. This study reports a positive and significant correlation between cortisol and waist/hip ratio (WHR) alone, WHR being a surrogate marker for abdominal obesity. Albeit, it has been suggested that a critical factor in the association between obesity, dyslipidaemia, hypertension, type 2 diabetes and cardiovascular morbidity, features of metabolic syndrome, is the intraabdominal fat mass [3]. Cortisol hyperactivity produces features of metabolic syndrome mediated through an enlarged visceral fat mass, cortisol being a powerful stimulator of visceral fat accumulation by its effect on lipoprotein lipase receptors which are highly concentrated in visceral adipose tissue [8, 37]. The expanded abdominal fat mass will result in insulin resistance through increased production of the adipokine, resistin [38]. Resistance to the actions of insulin on glucose and carbohydrate metabolism will give rise to the classical features of metabolic syndrome [39]. Cortisol also potentiates the lipolytic action of the β adrenergic receptors which also are concentrated in visceral adipose tissue, increasing free fatty acid flux and promoting insulin resistance [40]. Individuals with growth hormone deficiency have central obesity, insulin resistance atherosclerosis and increased mortality from cardiovascular diseases resulting both from growth hormone's pivotal role in lipid degradation and energy metabolism [3] and from its effect on cortisol metabolism [12]. In this study, growth hormone correlated positively with HDL cholesterol and negatively with diastolic blood pressure. Some studies have reported dyslipidaemias in individuals with growth hormone deficiency with increased total cholesterol, LDL- cholesterol, triglyceride and decreased HDL- cholesterol [41, 42]. Improvement in lipid parameters and diastolic blood pressure have also been recorded after growth hormone therapy in patients with growth hormone deficiency [3].

## Conclusion

Lower levels of T3 were associated with metabolic syndrome and its components in Nigerians. The finding of similar cortisol and growth hormone levels in metabolic syndrome and in healthy controls does not support a major role for cortisol and growth hormone in the pathogenesis of metabolic syndrome in Nigerians. The relationship of these hormones with components of the syndrome however suggests the possibility of a therapeutic role for these hormones in the management of metabolic syndrome. Interventional studies are therefore needed to further evaluate these relationships.

### What is known about this topic

- Endocrinopathies involving cortisol, growth hormone and the thyroid gland produce features of metabolic syndrome.

### What this study adds

- In adult Nigerians, individuals with metabolic syndrome have abnormalities of thyroid hormone secretion consistent with lower free T3 and higher free T4 concentrations than individuals without metabolic syndrome;
- In adult Nigerians with metabolic syndrome, serum levels of cortisol, thyroid and growth hormone correlate with components of metabolic syndrome and may suggest a therapeutic role for these hormones in the management of the syndrome.

## References

[1] Anagnostis P, Athyros VG, Tziomalos K, Karagiannis A, Mikhailidis DP (2009). Clinical review: the pathogenetic role of cortisol in the metabolic syndrome: a hypothesis. The J of Clin Endocrinol and Metabo..

[2] Uzunlulu M, Yorulmaz E, Oguz A (2007). Prevalence of subclinical hypothyroidism in patients with metabolic syndrome. Endocrin J..

[3] Johannsson G, Bengtsson BA (1999). Growth hormone and the metabolic syndrome. J Endocrinol..

[4] Bertagna X, Pinchera A (2001). Cushing's syndrome. Endocrinol and metabolism..

[5] Lacroix A, Pinchera A (2001). The adrenal cortex: basic concepts and diagnostic procedures. Endocrinology and metabolism..

[6] Swenson L, Knodel DH (2005). Adrenal function, Clinical Chemistry. Philadelphia Lippincott Williams and Wilkins..

[7] Kissebah A, Vydelingum N, Murray R, Evans D, Hartz A, Kalkhoff R (1982). Relationship of body fat distribution to metabolic complications of obesity. J Clin Endocrinol Metab..

[8] Mårin P, Darin N, Amemiya T, Andersson B, Jern S, Björntorp P (1992). Cortisol secretion in relation to body fat distribution in obese premenopausal women. Metabolism..

[9] Andrew R, Gale CR, Walker BR, Seckl JR, Martyn CN (2002). Glucocorticoid metabolism and the metabolic syndrome: associations in an elderly cohort. Exp Clin Endocrinol Diabetes..

[10] Andrews RC, Herlihy O, Livingstone DEW, Andrew R, Walker BR (2002). Abnormal cortisol metabolism and tissue sensitivity to cortisol in patients with glucose tolerance. J Clin Endocrinol Metab..

[11] Casanueva FF (1992). Physiology of growth hormone secretion and action. Endocrinol Metab Clin North Am..

[12] Stewart PM, Toogood AA, Tomlinson JW (2001). Growth hormone, insulin-like growth factor-I and the cortisol-cortisone shuttle. Horm Res..

[13] Udenze IC, Nnaji I, Oshodi TA (2014). Thyroid function in adult Nigerians with metabolic syndrome. Pan Afr Med J..

[14] Executive Summary of the Third report of the National Cholesterol Education Program (NCEP) (2001). Expert Panel on detection, evaluation, and treatment of high blood cholesterol in adults (Adult Treatment Panel III). JAMA..

[15] Adegoke OA, Adedoyin RA, Balogun MO, Adebayo RA, Bisiriyu LA, Salawu AA (2010). Prevalence of metabolic syndrome in a rural community in Nigeria. Metab Synd and Metab..

[16] Udenze IC, Azinge EC, Arikawe AP Egbuagha EU, Onyenekwu C, Ayodele O, Adizua UC (2013). The prevalence of metabolic syndrome in persons with type 2 diabetes at the Lagos University Teaching Hospital, Lagos Nigeria. WAJM..

[17] Malik S, Wong ND, Franklin SS (2004). Impact of the metabolic syndrome on mortality from coronary heart disease, cardiovascular disease, and all causes in United States adults. Circulation..

[18] Udenze IC, Azinge EC, Ebuehi OAT, Awolola NA, Adekola OO, Menkiti I (2012). The relationship between microalbuminuria, cardiovascular risk factors and disease management in type 2 diabetes. Nig Q J Hosp Med..

[19] Dandona P, Rosenberg MT (2010). A practical guide to male hypogonadism in the primary care setting. Int J Clin Pract..

[20] Schwartz SL, Rendell M, Ahmann AJ, Thomas A, Arauz-Pacheco CJ, Welles BR (2008). Safety profile and metabolic effects of 14 days of treatment with DIO-902: results of a phase IIa multicenter, randomized, double-blind, placebo-controlled, parallel-group trial in patients with type 2 diabetes mellitus. Clin Ther..

[21] World Health Organization (WHO) (2011). Waist circumference and waist-hip ratio: report of a WHO expert consultation. Circulation..

[22] Pickering TG, Hall JE, Appel LJ, Falkner BE, Graves J, Hill MN (2005). Recommendations for blood pressure measurement in humans and experimental animals. Circulation..

[23] Wild D (1996). Immunoassay Handbook..

[24] Ogbera OA, Kuku S, Dada O (2012). The metabolic syndrome in thyroid disease: A report from Nigeria. Indian J Endocrinol Metab..

[25] Iwen KA, Schröder E, Brabant G (2013). Thyroid Hormones and the Metabolic Syndrome. Eur Thyroid J..

[26] Biondi B (2010). Thyroid and Obesity: An Intriguing Relationship. The Jour of Clin Endocrinol & Metabo..

[27] Zimmermann-Belsing T, Brabant G, Holst JJ, Feldt-Rasmussen U (2003). Circulating leptin and thyroid dysfunction. Eur J Endocrinol..

[28] De Pergola G, Ciampolillo A, Paolotti S, Trerotoli P, Giorgino R (2007). Free triiodothyronine and thyroid stimulating hormone are directly associated with waist circumference, independently of insulin resistance, metabolic parameters and blood pressure in overweight and obese women. Clin Endocrinol (Oxf)..

[29] Bakker SJ, ter Maaten JC, Popp-Snijders C, Slaets JP, Heine RJ, Gans RO (2001). The relationship between thyrotropin and low density lipoprotein cholesterol is modified by insulin sensitivity in healthy euthyroid subjects. J Clin Endocrinol Metab..

[30] Bakker O, Hudig F, Meijssen S, Wiersinga WM (1998). Effects of triiodothyronine and amiodarone on the promoter of the human LDL receptor gene. Biochem Biophys Res Commun..

[31] Shin DJ, Osborne TF (2003). Thyroid hormone regulation and cholesterol metabolism are connected through Sterol Regulatory Element-Binding Protein-2 (SREBP-2). J Biol Chem..

[32] Prieur X, Huby T, Coste H, Schaap FG, Chapman MJ, Rodriguez JC (2005). Thyroid hormone regulates the hypotriglyceridemic gene APOA5. J Biol Chem..

[33] Rensen PC, van Dijk KW, Havekes LM (2005). Apolipoprotein AV: low concentration, high impact. Arterioscler Thromb Vasc Biol..

[34] Lagrost L (1994). Regulation of cholesteryl ester transfer protein (CETP) activity: review of in vitro and in vivo studies. Biochim Biophys Acta..

[35] Guzzetti C, Pilia S, Ibba A, Loche S (2014). Correlation between cortisol and components of the metabolic syndrome in obese children and adolescents. J of Endocrinol Invest..

[36] Ward AMV, Fall CHD, Stein CE, Kumaran K, Veena SR, Wood PJ (2003). Cortisol and the metabolic syndrome in South Asians. Clin Endocrinol (Oxf)..

[37] Peeke PM, Chrousos GP (1995). Hypercortisolism and obesity. Ann N Y Acad Sci..

[38] Steppan CM, Bailey ST, Bhat S (2001). The hormone resistin links obesity to diabetes. Nature..

[39] Reaven GM (1988). Banting lecture 1988: Role of insulin resistance in human disease. Diabetes..

[40] Arner P (1996). Regulation of lipolysis in fat cells. Diabetes Rev..

[41] Cuneo RC, Salomon F, Watts GP, Hesp R, Sonsken PH (1993). Growth hormone treatment improves serum lipids and lipoproteins in adults with growth hormone deficiency. Metabolism..

[42] Al-Shoumer KAS, Cox KH, Hughes CL, Richmond W, Johnston D (1997). Fasting and postprandial lipid abnormalities in hypopituitary women receiving conventional replacement therapy. J Clin Endocrinol Metab..

